# Long‐Term Trends in Prescribing of Etizolam for Hypnotic Use in a Japanese University Hospital

**DOI:** 10.1002/hup.70053

**Published:** 2026-06-23

**Authors:** Mutsuhiro Nakao, Hokuto Morohoshi, Yumiko Kurata, Akiou Nakamura

**Affiliations:** ^1^ Institute of Stress Management Showa Medical University Tokyo Japan; ^2^ Graduate School of Medicine Department of Hygiene Public Health and Preventive Medicine Showa Medical University Tokyo Japan; ^3^ Centre for Information Science and Technology & Department of General Emergency Medicine, Northern Yokohama Hospital Showa Medical University Tokyo Japan

**Keywords:** benzodiazepines, electronic medical records, etizolam, hypnotics, prescription trends, Z‐drugs

## Abstract

**Objectives:**

Etizolam is a short‐acting thienodiazepine widely prescribed for anxiety and insomnia despite increasing concerns regarding dependence and guideline recommendations limiting long‐term benzodiazepine use. This study examined long‐term prescribing trends of etizolam compared with benzodiazepine and non‐benzodiazepine hypnotics.

**Methods:**

We conducted a retrospective database study including 16,886,524 prescriptions issued between April 2001 and March 2022 at a tertiary university hospital in Japan. Seventeen hypnotic agents were identified (10 benzodiazepines and 7 non‐benzodiazepines). Multivariable logistic regression was used to identify factors associated with prescribing hypnotic etizolam versus non‐benzodiazepine hypnotics.

**Results:**

Among 124,179 etizolam prescriptions, 83,927 (67.6%) were issued for hypnotic use. Although the absolute number of prescriptions declined over time, the proportion prescribed for hypnotic use remained stable. The ratio of hypnotic etizolam prescriptions to non‐benzodiazepine hypnotics was higher in internal medicine than in psychiatry. In multivariable analysis, prescribing hypnotic etizolam was independently associated with internal medicine (adjusted OR 1.57), female sex, outpatient status, younger age, and earlier calendar year.

**Conclusions:**

Despite declining overall use, etizolam continues to be prescribed as a hypnotic, particularly in non‐psychiatric settings. These findings highlight the persistence of specialty‐specific prescribing patterns and underscore the importance of targeted educational and policy interventions to promote safer hypnotic prescribing practices.

## 1 Introduction

Etizolam is a benzodiazepine‐related thienodiazepine widely prescribed for both anxiolytic and hypnotic purposes (Nielsen and McAuley 2020; Jie et al. 2024), despite increasing concerns regarding dependence and guideline recommendations limiting long‐term use. Its continued use outside psychiatric settings and persistence following regulatory interventions raise important clinical and policy questions (Keenan et al. 2024; Yamagishi et al. 2025; Darke et al. 2026).

Clinical guidelines issued by organizations such as the Japanese Society of Sleep Research emphasize the risks associated with long‐term use, including dependence, falls and fractures in older adults, and potential cognitive impairment (Takaesu et al. 2023). Furthermore, prescribing guidelines targeting older adults have identified the addition of etizolam for sleep initiation—often contributing to polypharmacy—as a practice warranting reconsideration (Nishii et al. 2014; Tamburin et al. 2020; Kobayashi et al. 2021).

In response to accumulating evidence and professional concern, regulatory measures have been implemented in Japan. In 2016, the prescription duration for etizolam was, in principle, restricted to a maximum of 30 days Furthermore, the 2018 revision of the national medical fee schedule introduced reimbursement reductions for prescriptions continued at the same dosage for more than 1 year, thereby creating financial disincentives for prolonged use (Japan Pharmaceutical Information Centre 2025). Despite these regulatory and guideline‐based efforts, long‐term temporal trends in etizolam prescribing relative to other benzodiazepines and newer non‐benzodiazepine hypnotics have not been fully characterised: in particular, specialty‐specific prescribing patterns over extended periods remain unclear.

Etizolam warrants particular attention because of its distinctive prescribing profile. Unlike many other hypnotics, it is frequently prescribed in non‐psychiatric settings and often used concurrently for both anxiolytic and hypnotic purposes. In addition, its continued use despite regulatory restrictions suggests that prescribing behaviour may be influenced not only by clinical indications but also by specialty‐specific practices and familiarity with newer agents. These characteristics make etizolam a useful model for examining how prescribing patterns evolve in response to policy interventions. Differences in prescribing practices across specialties may be anticipated due to variations in clinical roles, training background, and familiarity with treatment guidelines. For example, psychiatrists may be more accustomed to guideline‐based prescribing of hypnotics, whereas physicians in other specialties may prioritise short‐term symptom management. Understanding these differences is important for identifying targets for intervention.

This study therefore addresses an important gap in understanding how prescribing practices evolve in response to both regulatory measures and clinical practice patterns, and aimed to characterise 21‐year trends in etizolam prescribing for hypnotic use compared with other benzodiazepine and non‐benzodiazepine hypnotics at a tertiary university hospital in Japan. We hypothesized that overall etizolam prescribing would decline following regulatory reforms, while relative use for hypnotic purposes and interdepartmental variation might persist.

## 2 Methods

### 2.1 Study Setting

This retrospective database study included all prescriptions issued between 1 April 2001 and 31 March 2022 at a tertiary university hospital located in a metropolitan area of Japan. The hospital has 689 licensed beds and provides care to approximately 60,000–70,000 patients annually. All clinical activities, including prescribing, were managed through a hospital‐wide computerized electronic medical record (EMR) system throughout the study period. Consequently, complete prescription data were available, and no missing prescription records were identified (Sundermann et al. 2025).

During the study period, 23 clinical departments were categorized into four groups: internal medicine (general internal medicine, respiratory medicine, gastroenterology, cardiology, endocrinology, and palliative care), psychiatry (general psychiatry), surgery (general surgery, neurosurgery, cardiac surgery, thyroid surgery, orthopaedics, plastic surgery, obstetrics and gynaecology, ophthalmology, otorhinolaryngology, dermatology, urology, oral surgery, and anaesthesia), and others (paediatrics, radiology, and emergency medicine).

A total of 1,305,506 patients were registered during the study period, accounting for 15,589,000 outpatient visits and 1,579,000 hospital admissions.

In the internal medicine group, the most frequent International Classification of Diseases, 10th Revision (ICD‐10) diagnoses were angina pectoris (6.7%), acute upper respiratory infections (5.6%), bronchitis (4.7%), gastric ulcer (4.5%), acute gastroenteritis (4.2%), hypertension (2.9%), diabetes mellitus (2.5%), constipation (2.4%), bronchial asthma (2.4%), and tension‐type headache (2.2%). In the psychiatry group, the most frequent diagnoses were major depressive disorder (29.5%), schizophrenia (11.3%), anxiety disorders (9.8%), bipolar disorder (5.9%), epilepsy (5.1%), adjustment disorder (3.8%), delirium (3.2%), eating disorders (1.8%), somatic symptom disorders (1.7%), and alcohol dependence (1.6%). In the surgery group, a variety of diseases and injuries were included like chronic gastritis (0.7%), lumbar spinal stenosis (0.6%), colorectal cancer (0.5%), and exertional angina (0.4%).

### 2.2 Prescriptions Assessed

Ten benzodiazepine hypnotics (brotizolam, estazolam, flunitrazepam, flurazepam, haloxazolam, lormetazepam, nitrazepam, quazepam, rilmazafone hydrochloride, and triazolam) were approved for use in Japan during the study period (Japan Pharmaceutical Information Centre 2025). Although etizolam is approved as both an anxiolytic and a hypnotic, it was classified as a benzodiazepine hypnotic when prescribed for bedtime administration and excluded when prescribed solely for daytime use. All benzodiazepine hypnotics included in the analysis had been approved before 2000.

Three Z‐drugs (eszopiclone [2012], zolpidem [2000], and zopiclone [1989]) and four newer non‐benzodiazepine agents (lemborexant [2020], melatonin [2020], ramelteon [2010], and suvorexant [2014]) were prescribed at the study hospital (years in brackets indicate approval in Japan). Prescriptions for these seven agents were categorized as non‐benzodiazepine hypnotics and were compared with prescriptions for etizolam and other benzodiazepine hypnotics.

### 2.3 Data Analysis

All analyses were performed using SAS statistical software (SAS Institute Inc. 2023). The unit of analysis was the prescription. Multiple prescriptions for the same patient were treated as independent observations, as the primary objective was to describe prescribing patterns at the prescription level rather than patient‐level treatment trajectories. This approach may introduce clustering effects and should be considered when interpreting the results.

Ratios of prescriptions for hypnotic etizolam to those for non‐benzodiazepine hypnotics were calculated according to department group, age group, sex, hospitalization status, and 5‐year periods. The final year (1 April 2021 and 31 March 2022) was analysed separately because it represented a single year. Non‐benzodiazepine hypnotics were selected as the primary comparator because they are increasingly recommended as alternatives to benzodiazepines in clinical guidelines and have been more recently introduced into clinical practice. Comparing etizolam with these agents provides insight into the extent to which prescribing patterns have shifted towards guideline‐preferred treatments.

Multivariable logistic regression analysis was conducted to examine factors associated with the prescription of hypnotic etizolam versus non‐benzodiazepine hypnotics, with particular focus on differences between internal medicine and psychiatry. The dependent variable was prescription of hypnotic etizolam (vs. non‐benzodiazepine hypnotics). Independent variables included department group (internal medicine vs. psychiatry), patient age (continuous, in years), sex (female vs. male), hospitalization status (inpatient vs. outpatient), and calendar year (continuous). Adjusted odds ratios (ORs) with 95% confidence intervals (CIs) were estimated. A two‐sided *p* value < 0.05 was considered statistically significant.

This study was approved by the university ethics committee (approval no. 22‐038‐A) and was conducted in accordance with institutional guidelines.

## 3 Results

### 3.1 Prescriptions of Etizolam

**FIGURE 1:**
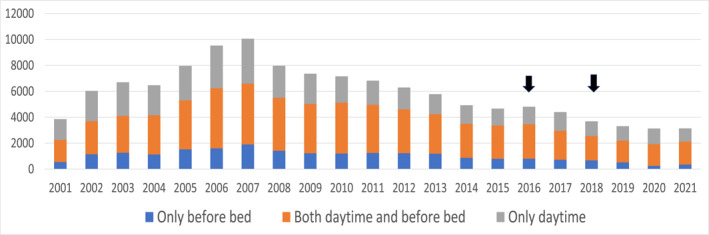
Annual number of etizolam prescriptions by usage category (2001–2021). The absolute number of etizolam prescriptions (*y*‐axis) is plotted against calendar year (*x*‐axis), stratified by usage category. Downward arrows indicate major regulatory interventions in Japan: the 2016 restriction limiting etizolam prescriptions to 30 days and the 2018 reimbursement revision targeting long‐term continuous prescribing.

Etizolam accounted for 0.74% of all prescriptions (124,179/16,886,524) issued during the study period (Table 1). Among etizolam prescriptions, 21,630 (17.4%) were for bedtime use only, 62,297 (50.2%) were prescribed both at bedtime and during the daytime, and 40,252 (32.4%) were prescribed for daytime use only. Overall, 83,927 prescriptions (67.6%) involved bedtime (hypnotic) use. The annual number of prescriptions of etizolam is shown in Figure 1. It peaked in 2007, and then gradually decreased until 2021.

**TABLE 1:** Prescription of etizolam according to usage in a university hospital.

	Usage including hypnotics			
Variables (the number of etizolam prescriptions)	Only before bed	Both daytime and before bed	Only daytime	Hypnotic prescription proportion^a^
Department group				
Internal medicine (*n* = 51,170)	9094 (17.8%)	29,196 (57.1%)	12,880 (25.2%)	0.748 [0.745, 0.752]
Psychiatry (*n* = 42,231)	8595 (20.4%)	15,174 (35.9%)	18,462 (43.7%)	0.563 [0.558, 0.568]
Surgery and related (*n* = 22,701)	3120 (13.7%)	13,064 (57.6%)	6517 (28.7%)	0.713 [0.707, 0.719]
Others (*n* = 8077)	821 (10.2%)	4863 (60.2%)	2393 (29.6%)	0.705 [0.696, 0.719]
Age group, years old				
< 15 (*n* = 65)	2 (3.1%)	18 (27.7%)	45 (69.2%)	0.308 [0.195, 0.420]
15–65 (*n* = 62,778)	11,168 (17.8%)	26,084 (41.6%)	25,526 (40.7%)	0.593 [0.590, 0.597]
> 65 (*n* = 61,336)	10,460 (17.1%)	36,195 (59.0%)	14,681 (23.9%)	0.761 [0.757, 0.764]
Gender				
Women (*n* = 76,377)	13,541 (17.7%)	36,521 (47.8%)	26,315 (34.5%)	0.655 [0.652, 0.659]
Men (*n* = 47,802)	8089 (16.9%)	25,776 (53.9%)	13,937 (29.2%)	0.708 [0.704, 0.713]
Hospitalization				
Outpatients (*n* = 93,282)	17,219 (18.5%)	44,667 (47.9%)	31,396 (33.7%)	0.663 [0.660, 0.666]
Inpatients (*n* = 30,897)	4411 (14.3%)	17,630 (57.1%)	8856 (28.7%)	0.713 [0.708, 0.718]
5‐year course group				
2001–2005 (*n* = 31,043)	5608 (18.1%)	13,913 (44.8%)	11,522 (37.1%)	0.629 [0.623, 0.634]
2006–2010 (*n* = 42,114)	7350 (17.5%)	21,154 (50.2%)	13,610 (32.3%)	0.677 [0.672, 0.681]
2011–2015 (*n* = 28,524)	5341 (18.7%)	15,350 (53.8%)	7833 (27.5%)	0.725 [0.720, 0.731]
2016–2020 (*n* = 19,351)	2978 (15.4%)	10,111 (52.3%)	6262 (32.4%)	0.676 [0.670, 0.683]
2021 (*n* = 3147)	353 (11.2%)	1769 (56.2%)	1025 (32.6%)	0.674 [0.658, 0.691]
Total (*n* = 124,179)	21,630 (17.42%)	62,297 (50.2%)	40,252 (32.4%)	0.676 [0.673, 0.678]

^a^ Proportion of prescriptions involving bedtime use (including combined daytime and bedtime use) to all etizolam prescriptions.

As shown in Table 2, the absolute number of prescriptions for hypnotic etizolam was highest in internal medicine (*n* = 38,000), followed by psychiatry (*n* = 24,000), surgery and related departments (*n* = 16,000), and other departments (*n* = 5700). Over time, the number of prescriptions for hypnotic etizolam declined after 2016, with approximately 13,000 prescriptions during 2016–2020 and 2100 in 2021, compared with 20,000–29,000 prescriptions per 5‐year period during 2001–2015.

**TABLE 2:** Prescription of hypnotics of etizolam and other benzodiazepines in comparison of non‐benzodiazepine hypnotics in a university hospital.

	Benzodiazepine hypnotics			
Variables (the number of prescriptions of hypnotics)	Etizolam as hypnotics	All benzodiazepines excluding etizolam	Non‐benzodiazepine hypnotics	The etizolam/non‐benzodiazepine ratio^a^
Department group				
Internal medicine (*n* = 246,590)	38,290 (15.5%)	89,597 (36.3%)	118,703 (48.1%)	0.323 [0.320, 0.325]
Psychiatry (*n* = 355,318)	23,769 (6.7%)	222,027 (62.5%)	109,522 (30.8%)	0.217 [0.215, 0.219]
Surgery and related (*n* = 71,281)	16,184 (22.7%)	20,170 (28.3%)	34,927 (49.0%)	0.463 [0.458, 0.469]
Others (*n* = 53,335)	5684 (10.7%)	15,889 (29.8%)	31,762 (59.6%)	0.179 [0.176, 0.182]
Age group, years old				
< 15 (*n* = 1337)	20 (1.5%)	518 (38.7%)	799 (59.8%)	0.025 [0.014, 0.036]
15–65 (*n* = 366,896)	37,252 (10.2%)	201,811 (55.0%)	127,833 (34.8%)	0.291 [0.289, 0.294]
> 65 (*n* = 358,291)	46,655 (13.0%)	145,354 (40.6%)	166,282 (46.4%)	0.281 [0.278, 0.283]
Gender				
Women (*n* = 425,862)	50,062 (11.8%)	208,041 (48.9%)	167,759 (39.4%)	0.298 [0.296, 0.301]
Men (*n* = 300,662)	33,865 (11.3%)	139,642 (46.4%)	127,155 (42.3%)	0.266 [0.264, 0.269]
Hospitalization				
Outpatients (*n* = 466,506)	61,886 (13.3%)	244,762 (52.5%)	159,858 (34.3%)	0.387 [0.385, 0.390]
Inpatients (*n* = 260,018)	22,041 (8.5%)	102,921 (39.6%)	135,056 (51.9%)	0.163 [0.161, 0.165]
5‐year course group				
2001–2005 (*n* = 151,422)	19,521 (12.9%)	76,168 (50.3%)	55,733 (36.8%)	0.350 [0.346, 0.354]
2006–2010 (*n* = 193,572)	28,504 (14.7%)	100,793 (52.1%)	64,275 (33.2%)	0.443 [0.440, 0.447]
2011–2015 (*n* = 158,621)	20,691 (13.0%)	82,115 (51.8%)	55,815 (35.2%)	0.371 [0.367, 0.375]
2016–2020 (*n* = 182,925)	13,089 (7.2%)	76,939 (42.1%)	92,897 (50.8%)	0.141 [0.139, 0.143]
2021 (*n* = 39,984)	2122 (5.3%)	11,668 (29.2%)	26,194 (65.5%)	0.081 [0.078, 0.084]
Total (*n* = 726,524)	83,927 (11.6%)	347,683 (47.9%)	294,914 (40.6%)	0.285 [0.283, 0.286]

^a^ Ratio of hypnotic etizolam prescriptions to non‐benzodiazepine hypnotic prescriptions.

### 3.2 Prescriptions of Other Benzodiazepines and Non‐Benzodiazepines

Excluding etizolam, brotizolam (*n* = 149,184) was the most frequently prescribed benzodiazepine hypnotic, followed by nitrazepam (*n* = 78,054), triazolam (*n* = 45,672), lormetazepam (*n* = 25,194), flunitrazepam (*n* = 23,240), and quazepam (*n* = 15,058) (all > 10,000 prescriptions). The total number of prescriptions for benzodiazepine hypnotics other than etizolam was approximately 60% lower in internal medicine than in psychiatry. In contrast, prescriptions for hypnotic etizolam were approximately 1.6‐fold higher in internal medicine than in psychiatry. Between 2001 and 2020, prescriptions for other benzodiazepine hypnotics ranged from 76,000 to 101,000 per 5‐year period, with a decrease observed in 2021 (*n* = 12,000).

Among non‐benzodiazepine agents, zolpidem (*n* = 168,463) was the most frequently prescribed, followed by suvorexant (*n* = 39,026), zopiclone (*n* = 34,932), eszopiclone (*n* = 25,220), and ramelteon (*n* = 20,805). The total number of non‐benzodiazepine prescriptions was comparable between internal medicine (*n* = 119,000) and psychiatry (*n* = 110,000). Although prescription volumes remained relatively stable between 2001 and 2015 (56,000–64,000 per 5‐year period), they increased during 2016–2020 (*n* = 93,000) and further in 2021 (*n* = 26,000).

### 3.3 Comparisons Between Hypnotic Etizolam and Non‐Benzodiazepine Hypnotics

**FIGURE 2:**
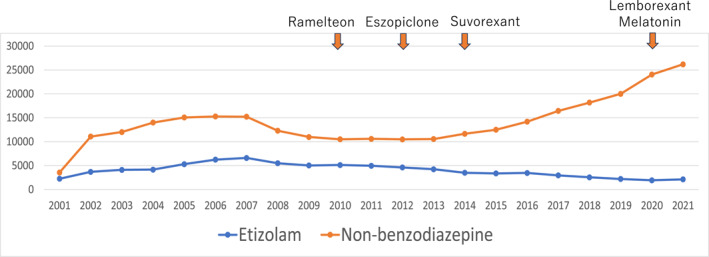
Annual number of prescriptions for etizolam and non‐benzodiazepine hypnotics (2001–2021). The absolute number of prescriptions for etizolam and non‐benzodiazepine hypnotics (*y*‐axis) is plotted against calendar year (*x*‐axis). Downward arrows indicate the approval years of major non‐benzodiazepine hypnotics introduced during the study period (ramelteon, eszopiclone, suvorexant, lemborexant, and melatonin).

The annual number of prescriptions of etizolam and non‐benzodiazepine hypnotics is shown in Figure 2. Overall, the ratio of prescriptions for hypnotic etizolam to those for non‐benzodiazepine hypnotics was 0.28 (= 0.2846) (Table 2). When stratified by department group, the ratio was highest in surgery and related departments (0.46) and lowest in psychiatry (0.22), excluding the “other” group (0.18).

Stratified analyses showed similar ratios between patients aged 15–65 years (0.29) and those aged ≥ 65 years (0.28). The ratio was higher in females (0.30) than in males (0.27), and higher in outpatients (0.39) than in inpatients (0.16).

Over time, the ratio declined markedly, from 0.35 to 0.47 during 2001–2015 to 0.14 during 2016–2020 and 0.08 in 2021.

Multivariable logistic regression analysis (Table 3) demonstrated that the odds of prescribing hypnotic etizolam rather than a non‐benzodiazepine hypnotic were significantly higher in internal medicine than in psychiatry, in females compared with males, and in outpatients compared with inpatients. Increasing patient age and later calendar year were independently associated with lower odds of etizolam prescribing.

**TABLE 3:** Factors discriminating the prescription of hypnotics for “etizolam (= 1)”^a^ or “non‐benzodiazepine (= 0)”^b^ in two departments of internal medicine and psychiatry: results of multiple logistic analysis.

Independent variables	Odds ratio
Internal medicine (= 1) or psychiatry (= 0)	1.573 [1.540, 1.606]
Age, years	0.983 [0.983, 0.984]
Female (= 1) or male (= 0) sex	1.239 [1.216, 1.263]
Outpatients (= 1) or inpatients (= 0)	2.347 [2.297, 2.397]
Calendar year	0.934 [0.932, 0.935]

^a^ The 21‐year cumulative number of hypnotic etizolam prescriptions was 62,059 (38,290 in internal medicine and 23,769 in psychiatry).

^b^ The 21‐year cumulative number of non‐benzodiazepine hypnotic prescriptions was 228,225 (118,703 in internal medicine and 109,522 in psychiatry).

## 4 Discussion

### 4.1 Time Trend of Etizolam Prescription

In this 21‐year database study, overall prescriptions of etizolam peaked in 2007 and subsequently declined through 2021. This decrease was observed across all patterns of use (daytime only, bedtime only, and combined use). However, the proportion of etizolam prescriptions involving hypnotic use remained relatively stable (63%–73%) throughout the study period.

In contrast, prescriptions for non‐benzodiazepine hypnotics remained stable until 2015 and increased thereafter. Consequently, the ratio of hypnotic etizolam prescriptions to non‐benzodiazepine hypnotics declined markedly after 2016. This temporal shift is consistent with evolving clinical guidelines and regulatory measures aimed at reducing benzodiazepine use (Takaesu et al. 2023). Although causal inference cannot be established in this observational study, the findings suggest that policy and guideline changes may have contributed to reductions in overall etizolam prescribing, while the relative ratio used for hypnotic purposes remained largely unchanged.

One possible explanation for the persistence of hypnotic use is that etizolam may continue to be perceived as a convenient short‐acting agent for sleep initiation, particularly in non‐specialist settings. In addition, its dual indication for anxiety and insomnia may encourage continued prescribing even when regulatory measures aim to reduce its use. These factors may contribute to the stability of the proportion of etizolam prescriptions involving hypnotic use despite reductions in overall prescribing volume.

### 4.2 Departmental Differences

A notable finding was the higher likelihood of prescribing hypnotic etizolam in internal medicine compared with psychiatry, even after adjustment for age, sex, hospitalization status, and calendar year. Although psychiatrists prescribed a greater overall number of benzodiazepine hypnotics other than etizolam, internists were more likely to prescribe etizolam specifically for hypnotic use.

This pattern may reflect differences in prescribing culture, training background, or familiarity with newer non‐benzodiazepine hypnotics (Nakao et al. 2006; Mansi et al. 2024). While newer agents—including Z‐drugs, ramelteon, suvorexant, and lemborexant—have been increasingly adopted, uptake may vary across specialties. These findings indicate that educational initiatives promoting evidence‐based hypnotic prescribing may be particularly relevant for non‐psychiatric departments (Okuda et al. 2023; Habukawa et al. 2024).

### 4.3 Prescribing in Older Adults

An important finding relates to prescribing patterns in older adults. Although benzodiazepine hypnotics other than etizolam were less frequently prescribed in older patients, hypnotic etizolam prescriptions remained comparatively common in this group. Notably, combined daytime and bedtime prescriptions accounted for a substantial ratio of etizolam use among older patients.

Given the well‐established risks of benzodiazepines in older adults—including falls, fractures, and cognitive impairment (Kobayashi et al. 2021; Rosenqvist et al. 2024)—continued combined daytime and hypnotic prescribing warrants careful evaluation. These findings underscore the importance of systematic medication review of long‐standing or overlapping etizolam prescriptions in elderly patients, particularly when prescribed for both anxiolytic and hypnotic purposes.

It is also important to consider healthcare system factors. Older adults are more likely to be managed in internal medicine rather than psychiatric settings, which may partly explain the higher prevalence of etizolam prescribing observed in this population. Differences in access to specialist care and patterns of healthcare utilisation may therefore contribute to the observed age‐related prescribing patterns.

### 4.4 Sex Differences

Female sex was independently associated with a higher likelihood of etizolam prescribing relative to non‐benzodiazepine hypnotics in the multivariable analysis. This finding is consistent with previous reports demonstrating higher benzodiazepine and etizolam use among women (Boyd et al. 2015; Tamburin et al. 2020; McIntyre et al. 2021; Milani et al. 2021; Liemburg et al. 2026).

Possible explanations include a higher prevalence of insomnia and anxiety disorders among women, as well as differences in healthcare‐seeking behaviour. For example, women may be more likely to seek care for sleep‐related complaints in primary care or internal medicine settings, which may influence prescribing patterns (McIntyre et al. 2021; Liemburg et al. 2026). These findings emphasize the need to ensure appropriate prescribing practices across sex groups, particularly in populations at increased risk of long‐term benzodiazepine exposure.

### 4.5 Outpatient Prescribing

Outpatient status was strongly associated with prescribing hypnotic etizolam rather than non‐benzodiazepine hypnotics. This may reflect more structured medication review and deprescribing efforts in inpatient settings, where closer monitoring and institutional safety protocols are in place.

International patient safety frameworks emphasize minimizing high‐risk medications, including benzodiazepines, particularly among elderly and postoperative patients (Evered and Pryor 2023; Shapoval et al. 2025). The lower relative use of etizolam in inpatient settings observed in this study is consistent with such risk‐reduction strategies.

### 4.6 Strengths and Limitations

This study has several limitations. First, it was conducted at a single tertiary university hospital, which may limit generalizability. Prescribing practices may differ in community hospitals or private clinics. Second, the analysis was based on prescription records and did not include clinical outcomes, treatment duration per patient, or dosage information. Repeated prescriptions from the same patient may have introduced clustering effects. Also, potential residual confounding may include unmeasured clinical severity, physician prescribing preferences, and institutional factors influencing medication selection. Accordingly, the reported associations should be interpreted as prescription‐level patterns rather than patient‐level risks. Third, the final study year (2021) coincided with the COVID‐19 pandemic in Japan, which may have influenced healthcare utilization and prescribing patterns.

However, this study also has important strengths. It represents one of the longest observational analyses of etizolam prescribing patterns in Japan (Okui et al. 2021), covering more than two decades and more than 16 million prescriptions. To our knowledge, no previous study has examined long‐term trends in etizolam prescribing relative to newer non‐benzodiazepine hypnotics using a large, integrated clinical database.

## 5 Conclusions

From an international perspective, etizolam use in Japan has been reported to be comparatively high (Kinoshita and Kishimoto 2023). Although regulatory and reimbursement reforms implemented between 2016 and 2018 aimed to promote appropriate psychotropic prescribing (Soumerai et al. 2024), the present findings suggest that, despite an overall decline in etizolam prescribing, specialty‐specific and outpatient prescribing patterns remain areas warranting attention.

Future strategies should prioritize targeted educational interventions in non‐psychiatric departments, systematic medication review in outpatient settings, and continued evaluation of policy measures to promote the appropriate use of hypnotics.

## Funding

This study was financially supported by Research Grant of the Japanese Ministry of Education, Culture, Sports, Science and Technology (23K09727) and the KDDI Foundation (2025‐MR‐003).

## Ethics Statement

This study was approved by the university ethics committee (approval no. 22‐038‐A).

## Consent

This study (opt‐out) was publicly disclosed on the university website in accordance with institutional policies.

## Conflicts of Interest

The authors declare no conflicts of interest.

## Permission to Reproduce Material From Other Sources

The authors have nothing to report.

## Data Availability

The data that support the findings of this study are available from the corresponding author upon reasonable request.
